# Age, gender, peers, life skills and quality of life influence risk of cell phone addiction among college teachers in Karnataka, India : a state level epidemiological analysis

**DOI:** 10.1186/s12889-022-12575-5

**Published:** 2022-01-26

**Authors:** BS Pradeep, Anusha B Shenoy, S Shahane, RN Srividya, Mutharaju Arelingaiah, Rochana D’Souza, Lavanya Garady, MK Jyoti, Suma Rache, Anand Dixit, Gananath Shetty Yekkar, Prathap Lingaiah, Shalini Rajneesh, G Gururaj

**Affiliations:** 1 Department of Epidemiology, Centre for Public Health, NIMHANS, Bengaluru, India; 2 Life Skills and Counselling Services Program, Department of Epidemiology, Centre for Public Health, NIMHANS, Bengaluru, India; 3 Department of Psychiatric Social Work, NIMHANS, Bengaluru, India; 4 Ramaiah International Centre for Public Health Innovations, Bengaluru, India; 5 CMR University Bengaluru, Bengaluru, India; 6 Department of Youth Empowerment and Sports, Government of Karnataka, Bengaluru, India; 7 Department of Epidemiology, Centre for Public Health, NIMHANS, Bengaluru, India

**Keywords:** Cell phone addiction, Technology addiction, Addictive behavior, Life skills, Quality of life

## Abstract

**Background:**

Cell phones are an integral part of modern day life and have become companions for individuals irrespective of age, gender and socio-economic status. In this study, we assessed the factors affecting risk of cell phone addiction among teachers attending Life Skills Training and Counselling Services (LSTCS) program in Karnataka.

**Methods:**

This cross sectional secondary data analysis utilised data from baseline assessment of trainees attending a Life Skills Training and Counselling Services program (LSTCP). Various factors hypothesised to be affecting risk of cell phone addiction (outcome) was analysed using univariate and multivariable logistic regression analysis. All the analysis was done using STATA 12.0 software.

**Results:**

Multivariable logistic regression analysis was conducted with risk of cell phone addiction as outcome. A conceptual framework of hypothesized exposure variables was developed based on expert consultation and literature review. Overall, data of 1981 participants was utilized. Gender (AOR=1.91; 95% CI=1.27-2.77), number of peers (AOR=1.01; 95 CI=1-1.008) and social quality of life (AOR=1.01; 95% CI=1.00-1.03) were associated with increased risk of cell phone addiction. Age (AOR=0.98; 95%CI=0.96-1.00), empathy (AOR=0.96;95%;CI=0.93-0.99), communication skills(AOR=0.92, 95%;CI=0.88-0.96) and physical quality of life (AOR=0.96; 95% CI=0.95-0.98) were associated with reduced risk of cell phone addiction.

**Conclusions:**

This study on precursors of risk of cell phone addiction, conducted mostly among apparently healthy individuals, provide important insights into interventions to reduce risk of cell phone addiction. The complexity of associations between peers, gender, quality of life and risk of cell phone addiction needs further exploration.

**Supplementary Information:**

The online version contains supplementary material available at 10.1186/s12889-022-12575-5.

## Background

Cell phones are an integral part of modern day life. There are about 5.2 billion unique cell phone users in the world [1]. As on 2019, there were about 1161.17 million cell phone users in India [2]. Cell phones are known to affect individuals overall health [3]. They are associated with sleep deprivation [4], inappropriate food habits [5], physical inactivity, over weight and obesity [6]. Further, reduced social participation, interaction with family, friends and society [7], road traffic accidents and injuries [8, 9] are associated with overuse cell phone and its addiction. In India, the magnitude of cell phone addiction among adolescents range from 39 to 44% [10]. Psychologically, cell phone use is attributed to loneliness, fatigue and stresses [11] and is a known precursor of consequent mental health problems [12].

Cell phones have become companions for individuals irrespective of age, gender and socio-economic status. This may lead to addiction amongst individuals. Various facets of cell phone addiction like “a state of socio-psychological illness”, “nomophobia” (No-Mobile-phobia) [13], “textiety”, “ringxiety”, “textaphrenia”, “phantom ringing/vibration syndrome”, “commufaking” are described. Approximately 2/3rd of the world’s population shows signs of nomophobia [14]. Excessive use of cellphone is also known to change brain chemistry [14]. Cell phone addiction is likely to affect an individual’s familial and societal relationships as they grow old and has potential to become a major public health problem [15]. College teachers are important and crucial change makers in the society as they have the responsibility in shaping life of youth and students. Education is no exception in contributing to the rapid growth of technology. Cellphones are known to impact education, health, social life and business [16]. These can both be positive and negative. Usage of cell phone among teachers includes potential obstacles such as student cheating, addiction to internet information, cyberbullying and negative impact on student conduct etc. Over use of cell phone results in ignoring day to day activities and disregard their responsibilities and commitments resulting in behavior addiction [17]. This impacts their quality of life [18], attention span [19], poor professional performance [20]. Cell phones kill creativity and conversations [21]. Improper use of cell phones during office can affect students negatively resulting in their poor academic performance, inability to efficiently complete assigned curriculum and increased pressure leading to decreased quality of life [22].

Understanding specific factors that contribute to cell phone addiction is imperative in order to plan strategies to minimize or eliminate those risks whenever possible and increasing quality of life. Further, this helps clinicians, public health professionals, policy makers and experts to work effectively towards the cause of cell phone addiction. Most studies have looked at cell phone addiction and its risk factors among adolescents and young adults. Current study focuses on identifying the various factors contributing towards developing risk of cell phone addiction which aids in the personal and professional growth. This study aims to address the issue of risk of cell phone addiction among college teachers attending Life Skills training and Counselling Services program (LSTCP) in Karnataka, India.

## Methods

This cross sectional secondary data analysis of various factors hypothesised to be affecting risk of cell phone addiction was conducted between January 2021 and March 2021. Secondary data from baseline assessment of trainees attending Life Skills Training and Counselling Services program (LSTCP) at National Institute of Mental Health and Neuro Sciences (NIMHANS), Bengaluru was utilised. Primarily the study was conducted to assess the effectiveness of Life skills training program among college teachers in Karnataka, India. The participants of LSTCP program are deputed mostly from within the government setup, namely directorates of collegiate education, technical education, pre-university board and 48 universities across 30 districts of Karnataka. Deputation of participants by their respective authorities is done on request by interested participants on a first-come-first-served basis. Data collection was done by trained project staff, where clear instructions provided before administration and participants’ questions were clarified during filling of responses. The primary data was collected using a pre-tested semi-structured self-administered pen and paper questionnaire, originally developed to assess effect of training on life skills of participants of LSTCP. This study instrument comprised of 25 sections (supplementary file 1). For this data analysis 10 out of these 25 sections namely socio-demographic details, sections on behaviour related to chewing and smoking tobacco, consuming alcohol, sniffing and injecting drugs, details of physical activity, information related to their occupation and peer characteristics, level of life skills and quality of life were utilised. Information on risk of cell phone addiction was utilised as outcome.

**Fig. 1:**
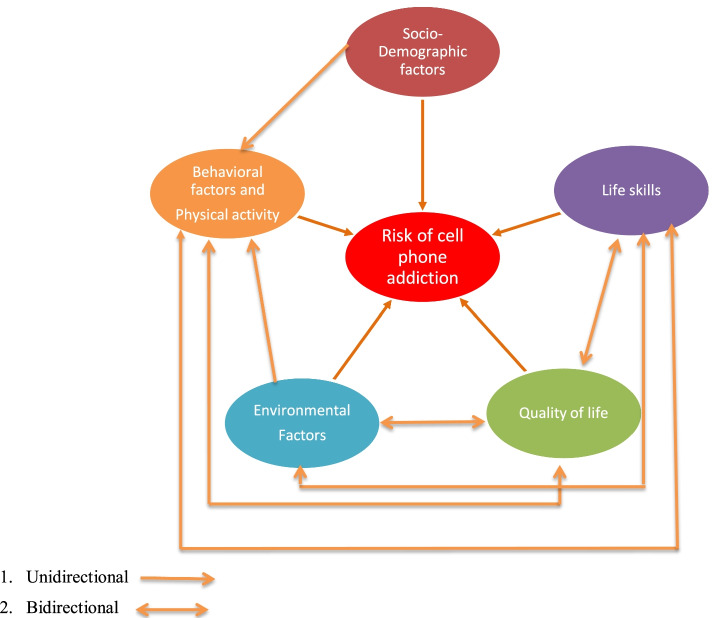
Conceptual framework of hypothesised factors associated with risk of cell phone addiction

Risk of cell phone addiction was assessed utilizing a 6-item questionnaire developed by the Centre for Well-Being NIMHANS, Bengaluru [7]. A conceptual frame work was developed depicting hypothesised exposure variables affecting risk of cell phone addiction (Fig. 1). A conceptual framework of factors affecting risk of cellphone addiction was developed based on stakeholder/expert consultation. These involved public health specialists, psychologists, psychiatrists, community development experts, teachers and youth. Broadly, these factors included socio-demographic factors, behavioural factors (chewing and smoking of tobacco, alcohol use, other substance use and personality traits), environmental factors (family environment, personal and family health, work and job satisfaction), individuals life skills score and quality of well-being scores.

### Statistical analysis

Univariate logistic regression analysis was performed with risk of cell phone addiction as outcome variable. Variables in the conceptual framework were considered as exposure variables. In univariate analysis, all hypothesised exposure variables associated with the outcome at 10% significance (p < 0.10) was eligible to be considered for the final multivariable logistic regression model. A forward stepping process was used to build the final model. Variables that were significant at 5% level (p < 0.05) and those which changed the odds ratio of at least one exposure variable by 10% were eligible to be retained in the final model. The significance of addition of each exposure variable into the model was tested using likelihood ratio test with appropriate degrees of freedom. This was done by comparing the nested model with the previous model. Goodness of fit for the final model was tested using *estat gof* command followed by fitting area under the curve using *lroc* command. All the analysis was done using STATA 12.0 software for WINDOWS [23]. All the necessary ethical guidelines and principles were followed in the conduct of this study. The ethical approval for the study was obtained from institutional ethics committee of NIMHANS vide letter No. NIMHANS/2^ND^ IEC (BS & NS DIV.)/2016 dated 07/12/2016. The primary data collection among participants of LSTCS program was done with written informed consent of the participants.

## Results

This study had 1981 participants. Among them, the majority were men (72.38%), mostly urban (59.99%), practiced hindu religion (92.67%), post graduates (89.16%) and currently married (77.17%). Mean age of participants at risk of cell phone addiction (37.82 ± 9.43 years) was significantly lower compared to those who were not at risk (39.67 ± 8.63) of cell phone addiction. Gender (p =0.001), education (p =0.039) and marital status (p =0.002) of participants was associated with risk of cell phone addiction (Table 1).

**Table 1:** Socio-demographic characteristics and risk of cell phone addiction among LSTCP participants

Socio demographic characteristics	Risk of cell phone addiction				Total		*p value
	Present		Absent				
	N	%	N	%	N	%	
**Age**^**$**^**(n=1927)**	37.82	9.43	39.67	8.63	39.44	8.76	0.003^¶^
**Gender (n=1937)**							
Female	42	7.85	493	92.15	535	27.62	0.001^¶^
Male	188	13.41	1214	86.59	1402	72.38	
**Locale (n=1937)**							
Rural	104	13.42	671	86.58	775	40.01	0.086
Urban	126	10.84	1036	89.16	1162	59.99	
**Religion (n=1937)**							
Hindu	216	12.03	1579	87.13	1795	92.67	0.44
Others	14	9.86	125	90.14	142	7.33	
**Education (n=1937)**							
Till PUC	4	16.67	20	83.33	24	1.24	0.039^¶^
Degree/Diploma	32	17.2	154	82.8	186	9.6	
PG and above	194	11.23	1533	88.77	1727	89.16	
**Marital status (n=1934)**							
Currently married	160	10.65	1343	89.35	1503	77.71	0.002^¶^
Never married	66	17.23	317	82.77	383	19.8	
Others	4	8.33	44	91.67	48	2.48	

^*^p value for chi-square test for independence for categorical variables/fisher’s exact test for categorical variables and t-test for difference between two means for continuous variables ^$^Numbers indicate Mean and Standard deviation in place of number and percentage, ^¶^significant at *p*<0.05

Majority of the participants reported to be involved in daily physical activity (85.07%). Among them almost 3/4th (87.62%) reported to be involved in moderate physical activity. About 20.72% (n = 357) of the participants reported to feel excessively anxious. More than half of the participants reported to engage in self-talk (57.32%) and about 4.29% reported of having suicidal thoughts. Approximately, 1/3rd of the participants reported to have consumed alcohol (29.04%) and 13.28% reported to have ever smoked. Daily physical activity, feeling depressed, feeling excessively anxious, participants who engage in self-talk, those who ever smoked, ever injected drugs to get high and personality traits such as extraversion, agreeableness, conscientiousness and neuroticism were all significantly associated with risk of cell phone addiction among the study participants (Table 2).

**Table 2:** Physical activity, behavioral factors, substance use characteristics and risk of cellphone addiction among LSTCP participants

Physical activity, behavioral factors and substance use	Risk of cell phone addiction						Total		*p* value*	
	Present			Absent						
	n	%		n	%		N	%		
**Physical activity (n = 1915)**										
Daily routine involves physical activity	181	11.11		1448	88.89		1629	85.07	0.01^¶^	
Type of physical activity (n = 1623)**										
Sedentary	20	15.5		109	84.5		129	7.95	0.249	
Moderate	152	10.69		1270	89.31		1422	87.62		
Vigorous	8	11.11		64	88.89		72	4.44		
**Behavioral characteristics /Psychological wellbeing**										
Ever smoked tobacco (n = 1928)	41	16.02		215	83.98		256	13.28	0.03^¶^	
Currently smoking tobacco (n = 226)	14	19.18		59	80.82		73	32.3	0.178	
Ever used smokeless tobacco (n = 1915)	14	15.05		79	84.95		93	4.86	0.346	
Currently using smokeless tobacco (n = 86)	3	10		27	90		30	34.88	0.529	
Ever consumed alcohol (n = 1911)	72	12.97		483	87.03		555	29.04	0.37	
Ever used injecting drugs to get high (n = 1909)	6	25		18	75		24	1.26	0.047^¶^	
Ever used sniffing drugs to get high (n = 1920)	3	21.43		11	78.57		14	0.73	0.226	
Feel depressed (n = 1588)	4	33.33		8	66.67		12	0.76	0.037^¶^	
Feel excessively anxious (n = 1723)	66	18.49		291	81.51		357	20.72	<0.001^¶^	
Suicidal ideation (n = 1912)	15	18.29		67	81.71		82	4.29	0.072	
Self-harm (n = 1828)	4	26.67		11	73.33		15	0.82	0.088	
Self-talk (n = 1912)	144	13.14		952	86.86		1096	57.32	0.047^¶^	
**Personality traits** ^$^										
	**Mean**	**SD**	**Mean**		**SD**	**Mean**		**SD**		**p**
Extraversion score (n = 1902)	3.29	0.8		3.51	0.78		3.49	0.78	<0.001^¶^	
Agreeableness score (n = 1906)	3.7	0.66		3.86	0.65		3.85	0.66	<0.001^¶^	
Conscientiousness score (n = 1909)	3.87	0.74		4.08	0.66		4.06	0.68	<0.001^¶^	
Neuroticism score (n = 1912)	2.44	0.79		2.14	0.72		2.17	0.73	<0.001^¶^	
Openness score (n = 1919)	3.13	0.45		3.13	0.42		3.13	0.42	0.84	

^*^p value of fisher’s exact test, chi-square test for independence for categorical variables/t-test for difference between two means for continuous variables, ^$^ Numbers indicate Mean and Standard deviation(SD) in place of number and percentage, **Among those participants whose daily routine involved physical activity, ^¶^significant at *p* < 0.05

Majority of the participants spend time with their family (96.07%), participate in regular picnics and social gatherings (95.23%), take collective decisions in family (76.54%) and have good family support (67.41%). Participation in social gatherings within the family, decision making within the family, job satisfaction, number of peers and having health problems were associated with risk of cell phone addiction among participants attending LSTCP (Table 3).

**Table 3:** Environmental factors and risk of cellphone addiction among LSTCP participants

Family and social characteristics	Risk of cell phone addiction				Total		*p* value*
	Present		Absent				
	N	%	N	%	N	%	
Spend time with family (n = 1934)	218	11.73	1640	88.27	1858	96.07	0.284
Participate in social gatherings with family (n = 1929)	210	11.43	1627	88.57	1837	95.23	0.003^¶^
**Decision making in family (n = 1931)**							
Collectively make decision	163	11.03	1315	88.97	1478	76.54	0.049^¶^
I make decision	56	15.64	302	84.36	358	18.54	
Somebody else make decision	10	10.53	85	89.47	95	4.92	
**Justification of arguments within the family (n = 1312)**							
Completely justified	34	11.93	251	88.07	285	21.72	0.175
Usually justified	60	10.95	488	89.05	548	41.77	
Sometimes justified	67	15.58	363	84.42	430	32.77	
Not at all justified	7	14.29	42	85.71	49	3.73	
**Family support (n = 1918)**							
Completely supportive	149	11.52	1144	88.48	1293	67.41	0.359
Usually supportive	55	11.96	405	88.04	460	23.98	
Sometimes supportive	20	13.99	123	86.01	143	7.46	
Not at all supportive	5	22.73	17	77.27	22	1.15	
**Participant has health related problems**	96	13.99	590	86.01	686	35.53	0.036^¶^
**Had been diagnosed with a health problem**							
Hypertension (n = 679)	29	13.24	190	86.76	219	32.25	0.803
Diabetes mellitus (n = 678)	23	15.23	128	84.77	151	22.27	0.2
Thyroid disorders (n = 678)	10	10	90	90	100	14.75	0.199
Rheumatic heart disease (n = 678)	6	23.08	20	76.92	26	3.83	0.23
Congenital heart disease (n = 678)	1	16.67	5	83.33	6	0.88	0.371
Other cardiac disorders (n = 678)	2	12.5	14	87.5	16	2.36	0.468
Stroke (n = 678)	0	0	7	100	7	1.03	0.647
Cancer (n = 678)	1	20	4	80	5	0.74	0.591
Mental health problems (n = 678)	13	16.46	66	83.54	79	11.65	0.369
**Job satisfaction (n = 1910)**							
Strongly satisfied	133	10.48	1136	89.52	1269	66.44	0.002^¶^
Satisfied	72	13.16	475	86.84	547	28.64	
Neither satisfied nor dissatisfied	17	22.67	58	77.33	75	3.93	
Dissatisfied	3	42.86	4	57.14	7	0.37	
Strongly dissatisfied	1	8.33	11	91.67	12	0.63	
Participants stay away from family for work (n = 1918)	146	12.67	1006	87.33	1152	60.06	0.138
Number of peers^ȵ^	50	85	25	85	25	85	0.019^¶^
Member of any groups, organization or association (n = 1899)	107	12.23	768	87.77	875	46.08	0.543

^*^p value of fisher’s exact test, chi-square test for independence for categorical variables, Mann-Whitney U test, ^ȵ^ Median with interquartile range, ^¶^significant at *p *< 0.05

Overall, increasing life skills scores and quality of life scores across domains were significantly associated with reduced risk of cell phone addiction except creative thinking scores (Table 4).

**Table 4:** Life skills, quality of life and risk of cell phone addiction among participants attending LSTCP

Life skills and quality of life	Risk of cell phone addiction				Total		Crude Odds ratio	Confidence interval at 95%	*p value
	Present		Absent						
	Mean	SD	Mean	SD	Mean	SD			
Decision making (n = 1937)	35.23	3.94	36.84	3.86	36.64	3.9	0.902	0.87-0.93	<0.001^¶^
Problem solving (n = 1937)	51.34	6.07	53.99	5.91	53.68	6.07	0.933	0.91-0.95	<0.001^¶^
Empathy(n = 1937)	45.35	5.55	48.36	5.49	48	5.58	0.911	0.89-0.93	<0.001^¶^
Self-awareness (n = 1937)	40.05	4.96	41.36	4.59	41.2	4.65	0.945	0.92-0.97	<0.001^¶^
Communication skills (n = 1937)	36.55	4.07	39.03	4.31	38.73	4.31	0.874	0.85-0.90	<0.001^¶^
Interpersonal relationship skills (n = 1937)	70.36	7.68	73.47	6.89	73.1	7.1	0.943	0.93-0.96	<0.001^¶^
Coping with emotions (n = 1937)	34.64	4.27	36.13	3.89	35.95	3.97	0.915	0.88-0.95	<0.001^¶^
Coping with stress (n = 1937)	34.13	4.28	34.73	4.06	34.66	4.09	0.966	0.93-0.99	<0.001^¶^
Creative Thinking (n = 1937)	54.38	7.5	54.07	7.07	54.1	7.12	1.006	0.99-1.03	0.533
Critical thinking (n = 1937)	38.7	5.49	39.44	4.91	39.35	4.98	0.971	0.95-0.99	0.034^¶^
Overall life skill score (n = 1937)	440.74	41.98	457.42	39.28	455.44	39.97	0.99	0.98-0.99	<0.001^¶^
Overall quality of life and health satisfaction (n = 1932)	8.05	1.18	8.28	1.08	8.25	1.1	0.835	0.74-0.94	0.003^¶^
Physical quality of life (n = 1917)	72.93	12.74	79.33	12.08	78.56	12.33	0.962	0.95-0.97	<0.001^¶^
Psychological quality of life (n = 1901)	68.79	12.63	71.01	10.73	70.74	10.99	0.982	0.97-0.99	0.005^¶^
Social quality of life (n = 1810)	75.89	16.51	78.84	15.61	78.48	15.74	0.989	0.98-0.99	0.01^¶^
Environmental quality of life (n = 1915)	67.46	13.57	71.77	13.03	71.25	13.17	0.976	0.97-0.98	<0.001^¶^

^*^p value for univariate logistic regression; ^¶^significant at *p* < 0.05; SD is Standard deviation

In multivariable logistic regression analysis, participant’s age and gender; number of peers the participant reported to be having; empathy and communication skills; physical and social quality of life were significantly associated with risk of cell phone addiction among participants of LSTCP (Table 5). For every unit increase in age, the odds of cell phone addiction decreased by 2% (AOR=0.98; 95%CI=0.96-1.00). Male participants had almost 2 times higher odds of cell phone addiction compared to female participants (AOR=1.91; 95% CI=1.27-2.77). Increase in number of peers was associated with increased odds of cell phone addiction (AOR=1.01; 95 CI=1-1.008). Among the different life skills domains every unit increase in empathy (AOR=0.96; 95% CI=0.93-0.99) and communication skills (AOR=0.92; 95% CI=0.88-0.96) was associated with 4% and 8% reduction in odds of cell phone addiction respectively. Every unit increase in physical quality of life (AOR=0.96; 95% CI=0.95-0.98) was associated with 4% reduction in odds of cell phone addiction while every unit increase in social quality of life score (AOR=1.01; 95% CI=1.00-1.03) was associated with 1% increased odds of cell phone addiction.

**Table 5:** Multiple logistic regression analysis of factors affecting risk of cell phone addiction among participants attending LSTCP (n = 1726)

Characteristics	Crude odds ratio	95% Confidence interval(CI)	*p* value^a^	Adjusted odds ratio(AOR)	95% CI	*p* value*
Age	0.98	0.96-0.99	0.003^b^	0.98	0.96-1.00	0.026^b^
Gender Female Male	Reference 1.82	Reference 1.28-2.58	Reference 0.001^b^	Reference 1.91	Reference 1.27-2.77	Reference 0.002^b^
Number of peers	1.005	1.001-1.008	0.007^b^	1.005	1-1.008	0.023^b^
Empathy score	0.91	0.89-0.93	<0.001^b^	0.96	0.93-0.99	0.012^b^
Communication skills score	0.87	0.85-0.93	<0.001^b^	0.92	0.88-0.96	<0.001^b^
Physical quality of life	0.96	0.95-0.97	<0.001^b^	0.96	0.95-0.98	<0.001^b^
Social quality of life	0.98	0.98-1	0.01^b^	1.01	1.00-1.03	0.014^b^

^a^crude and adjusted p value of univariate and multiple logistic regression and ^b^significant at *p* < 0.05

Goodness of fit (Area Under the Curve)=0.72 ; Hosmer lemeshow chi2=1661.36, *p* = 0.819

## Discussion

Our study throws light on the factors associated with risk of cell phone addiction among LSTCP participants. Gender, number of peers and social quality of life were associated with increased risk of cell phone addiction. Age; empathy; communication skills and physical quality of life were associated with reduced risk of cell phone addiction among participants of LSTCP (Table 5).

Younger individuals lack self-control and prudence for appropriate utilization of cellphones [24–27]. It is known that younger individuals are more tech savvy and comfortable using cell phones compared to older individuals. Similar to other studies, we report, decreased risk of cell phone addiction with age. In addition, reduced adaptability in advancement of cell phones may contribute to reduced usage and subsequent risk of cell phone addiction among older individuals. This might be the case with our study population of teachers. Association of gender with cell phone addiction is not consistent across studies [28]. In conformity with few studies, we found that risk of cell phone addiction is more among men compared to women [27, 29]. However, there are other studies which report either no difference in risk [30, 31] or increased risk among women [32]. There is a need to explore this inconsistent association of gender with risk of cell phone addiction.

In our study, increasing number of peers increased the risk of cell phone addiction. Better social quality of life was also associated with increased risk of cell phone addiction. This might be a reflection of increased interaction with peers through social media and instant messaging platforms (IMPs). It is likely for the participants to consider their contacts in social media and IMPs as peers. In addition, participants are likely to be utilising cell phones for their social interactions with those peers with whom in-person interaction was not possible. However, this information was not verified during data collection. As per our knowledge there is only one study related to peers and cell phone addiction inferring that peer satisfaction lowers the risk of cell phone addiction [33]. There are no studies looking at number of peers and cell phone addiction risk. It is likely that number of peers and peer satisfaction might be correlated. However, this data was not collected in our study.

Among the ten life skills domains, every unit increase in scores of empathy and communication skills reduced the risk of cell phone addiction by 4% and 8% respectively. As explained by Funk and Buchman, exposure to media and cyberspace influences the behaviour of individual [34]. Use of any gadget for long duration is known to have negative impact on empathy and vice versa [34]. Further, higher smart phone addiction score is known to have negative impact on interpersonal communication [35]. Logically, when there are people around to communicate and empathise with each other, the urge to use a cell phone will likely reduce with subsequent reduction in risk of cell phone addiction.

It is known that quality of life impacts negatively on risk of cell phone addiction among the young [36]. We found that physical quality of life significantly reduced the risk and social quality of life increased the risk of cell phone addiction. Another study [37] among adolescents showed negative correlation of physical, psychosocial and overall quality of life with smart phone addiction. The difference in age group of study population might be the reason for the contradictory results between these studies. In addition, difference in study instruments, sampling design and social contexts might affect these contradictory findings. The complexity associated with risk of cell phone addiction and different domains of quality of life emphasize the need to further examine these influences to inform interventions to improve quality of life.

The use of cell phones is becoming universal and an integral part in everyday life of individuals. This study comprehensively assessed 61 hypothesised factors associated with risk of cell phone addiction rather than factors associated with cell phone addiction. This provides an opportunity to intervene at a higher level in the pathway of development of cell phone addiction. Most studies on cell phone addiction have focused on adolescent and young population [10, 24, 36–38]. This study is conducted on largely adult population (mean age=39.44 ± 8.76), mostly literate, married and post graduates. The results of this study are generalizable only to this population and contribute to the existing knowledge related to cell phone addiction beyond adolescent and young population.

The risk of cell phone addiction was assessed using standardised and validated tool developed by the centre for wellbeing NIMHANS, Bengaluru. This tool is utilized routinely in clinical practice to detect risk of cell phone addiction. There are many tools to assess technology addiction namely game addiction [39, 40], smartphone addiction [30], television addiction [41], internet addiction [42, 43] etc. This study used secondary data of individuals attending LSTCP. Primarily, the data for LSTCP was collected to assess factors affecting life skills. This is a 57-page questionnaire with 25 sections. Thus, the NIMHANS centre for wellbeing scale on risk of cell phone addiction being a small 6-item questionnaire was included as a factor affecting life skills. The scales used to assess quality of life and life skills are both standardised and validated for use [44, 45]. The Big5 inventory utilized to assess personality traits of participants is also a standardised and validated tool for use among adult population [46]. Furthermore, the large sample size, participants from various districts across Karnataka and utilizing secondary data adds to the strengths of the results of the study.

### Limitations

The study is not without limitations. The participants of LSTCP program are deputed mostly from within the government setup, namely directorates of collegiate education, technical education, pre-university board and 48 universities across 30 districts of Karnataka. Although the selection of participants is on deputation, there is considerable geographic representation of participants from across the state. On an average there are approximately 66 participants deputed per district. We expect that these deputed officers are no different from those who are not deputed. Hence, we feel that the influence of selection bias related to outcome is either unlikely or negligible. However, to our knowledge, supporting evidence for the same is not available in current existing literature. Data collection using self-administered questionnaire offers limited control over the responses provided as well as the order in which respondent fills the questionnaire. However, the presence of one of our project team members to facilitate respondents, while filling the questionnaire as well as providing clear instruction and informed consent prior to questionnaire administration is likely to minimize this limitation. However, the data collection being self-administered, and training of project team is likely to ensure minimizing the effect of this bias and overall outcome. The presence of team member was also to clarify the doubts of the participants if they had any and there was no pressure/forcing on respondents for desirable answer in favor of the study. Highest level of control over the questionnaire was with the participants as it was a self-administered questionnaire reducing the interviewer and social desirability bias.

## Conclusion

Despite limitations, this study has important implications for researchers and practitioners working on health promotion related to technology or cell phone or internet addiction among adults especially teachers. This study, being focused on precursors of risk of cell phone addiction, conducted mostly among apparently healthy individuals provides important insights into interventions upstream. Health promotion programs related to cell phone use among teachers could utilise these findings while designing interventions. However, the complexity of associations between quality of life and risk of cell phone addiction, number of peers and risk of cell phone addiction, various aspects of peer involvement like quality of peer association, satisfaction with peers need further exploration.

## Supplementary Information

**Additional file 1.**

## Data Availability

The datasets used and/or analyzed during the current study are available from the corresponding author on reasonable request.

## Abbreviations

LSTCS/P: Life Skills Training and Counselling Services /Program
AOR: Adjusted odds ratio
CI: Confidence interval IMP: Instant messaging platform
IMP: Instant messaging platform
