# Supplementary material for: Age, gender, peers, life skills and quality of life influence risk of cell phone addiction among college teachers in Karnataka, India : a state level epidemiological analysis
Source: BMC Public Health. 2022 Jan 26;22:180. doi: 10.1186/s12889-022-12575-5 (PMC8793159; doi:10.1186/s12889-022-12575-5)
Supplement: Supplementary file 1 — Additional file 1. [file 12889_2022_12575_MOESM1_ESM.docx]

**Supplementary file 1**

The pen and paper self-administered questionnaire consisted of 25 sections. They are,

Section 1- Interview information: This includes unique study number, name, address, date and place of study along with consent information

Section 2 - Editing and data entry: contains information regarding data entry, name of the supervisor who collected the data, date of data collection and place of study where the data was collected along with interviewer codes and other related information, in addition to information related to the person who entered data along with time of data entry.

Section 3 - Socio-demographic characteristics: contains information on age, gender, religion, caste/tribe, education qualification and marital status. This section was linked to a household information sheet which had information about number of family members and details regarding their relationship with the respondent, their gender, age, occupation, education and marital status.

Section 4 - Family environment: comprised information about communication, arguments, and criticisms among family members. Further, it focused on the time spent with family members and decisions made in the family; and information regarding family support in terms of their perceived level of support from their family members.

Section 5 - Socio - economic characteristics: contained information about household monthly income, and monthly expenditure was collected.

Section 6 - Personal and family health: involved information on different forms of disease, injuries and hospitalization among self and family members including siblings of the respondents

Section 7 - Diet and eating habits: Contained information regarding type of diet consumed by the respondent along with frequency of consumption of food at home.

There was a separate section consisting of 13 screening questions with one question each to screen for tobacco smoking, chewing, alcohol consumption, injecting or oral drug use, use of sniffing drugs, violence, and injuries. There were two questions each to screen for depression, anxiety, and suicidality. Answering ‘Yes’ to any of these questions lead them to answer the appropriate section for detailed information. Section 8, 9, 10 and 11 contained information on tobacco smoking, chewing, alcohol consumption, and injecting/sniffing/oral drugs respectively. They contained information on age at start of use, current status of use and questionnaire to assess dependence. Tobacco smoking and injecting/sniffing/oral drug dependence was assessed using CAGE questionnaire (1). Tobacco chewing, and alcohol dependence was assessed using modified Fragerstorm questionnaire (2) and alcohol dependency scale (3).

Section 12 - Violence related information: this section consisted information on different forms of violence experienced by respondent, its frequency, the person who inflicted violence, hospitalization due to injuries and information on violence inflicted by the respondent to others

Section 13 - Depression: This contained general questions looking at screening for depression in which the questions were asked to seek information on symptoms of depression in the last 15 days like loss of appetite, sleep disturbance, feeling apathy, feeling worthless, lack of interest in daily activities and at work.

Section 14 - Generalized anxiety disorder: had information on symptoms of anxiety like consistently being worried, feeling restless, irritable, and inability to concentrate on work

Section 15 - Suicidality: had information related to having thoughts of committing self-harm or suicide, frequency, and intensity of such thoughts, ever attempted to commit suicide, and ability to control such impulses

Section 16 - Injuries and related: information on different forms of injuries and hospitalization due to the same. Injuries like road traffic accidents, falls, burns, animal bites, drowning and poisoning were included in this section

Section 17 - Physical activity: this section focused on time spent on different types of physical activities in a typical week. Activities were divided into vigorous, moderate, and sedentary physical activities based on WHO guidelines (4)

Section 18 - Sexual practice: here questions were related to participant’s sexual behavior including sexual practices, information on sexual activity, number of partners, use of condoms during first and the last time the person had sex.

Section 19 - Work environment & Job satisfaction: consisted of information related to work environment and job satisfaction, work related factors like current organization, change in job, work experience.

Section 20 - Teaching factors: factors related to their teaching like participant’s usual mode of teaching, preferred mode of teaching, perception about their teaching abilities, knowledge and technology assisted teaching

Section 21 - Peer group and social capital: this section included information on number of peers the participant had, activities they do with peers and information on peer characteristics.

Section 22 - Behavioral factors: information on self-talk, crisis (social, family, work related, financial, psychological, health related) in subjects’ life, and psychological factors. Also, Big Five Inventory- 10 (BFI-10) questions were asked to assess the personality type of the subject within this section. (5)

Section 23 - Life skills: There were 115 questions related to life skills measured on a 5-point likert rating scale including all the 10 domains of life skills as prescribed by WHO (6).

Section 24 - Quality of life: Questions regarding this section was measured by WHOQOL-BREF (7). It is an abbreviated 26 item version of the WHOQOL-100. The WHOQOL-BREF contains one item from each of the 24 facets of QOL included in the WHOQOL-100, plus two ‘benchmark’ items from the general facet of overall QOL and general health. The reliability, internal consistency, validity of the questionnaires was very good

Section 25 - Exposure to media and related: This section contained information with respect to television, internet, video tapes, video games, and mobile technology usage, with number of hours spent on them, type of use and kind of programs they watch in addition to the NIMHANS Centre for Well being scale to assess risk of cell phone addiction (8)

Reference:

1. Ewing JA. Detecting alcoholism: the CAGE questionnaire. Jama. 1984 Oct 12;252(14):1905-7.
2. Heatherton TF, Kozlowski LT, Frecker RC, Fagerstrom KO. The Fagerstrom test for nicotine dependence: a revision of the Fagerstrom Tolerance Questionnaire. British journal of addiction. 1991 Sep; 86(9):1119-27.
3. D. Sheehan JJ, Baker R, Harnett-Sheehan K, Knapp E, Sheehan M. Mini international neuropsychiatric interview (M.I.N.I.) English Version 6.0.0 ICD-10 USA: University of South Florida.
4. WHO W. Global recommendations on physical activity for health. Geneva World Heal Organ. 2010 Apr;60.
5. Rammstedt, B. & John, O. P. (2007). Measuring personality in one minute or less: A 10-item short version of the Big Five Inventory in English and German. Journal of Research in Personality, 41, 203-212.
6. Vranda MN. Development and standardization of life skills scale. Indian Journal of Social Psychiatry. 2009;25(1/2):17-28.
7. Whoqol Group. Development of the World Health Organization WHOQOL-BREF quality of life assessment. Psychological medicine. 1998 May;28(3):551-8.
8. NIMHANS Centre for Well-Being. Cell phone over use and addiction [available from: <https://nimhans.ac.in/wp-content/uploads/2019/02/Cell-phone-overuse-and-addiction.pdf>. Accessed on Feb – 2019
